# Impaired Cerebrospinal Fluid Lipoprotein-Mediated Cholesterol Delivery to Neurons in Alzheimer’s Disease

**DOI:** 10.21203/rs.3.rs-5682870/v1

**Published:** 2024-12-23

**Authors:** Carla Borràs, Marina Canyelles, David Santos, Noemí Rotllan, Estefanía Núñez, Jesús Vázquez, Daniel Maspoch, Mary Cano-Sarabia, Maria Carmona-Iragui, Sònia Sirisi, Alberto Lleó, Juan Fortea, Daniel Alcolea, Francisco Blanco-Vaca, Joan Carles Escolà-Gil, Mireia Tondo

**Affiliations:** Institut de Recerca Sant Pau; Hospital de la Santa Creu i Sant Pau; Centro de Investigación Biomédica en Red Diabetes y Enfermedades Metabólicas Asociadas; Institut de Recerca Sant Pau; Centro Nacional de Investigaciones Cardiovasculares Carlos III; Centro Nacional de Investigaciones Cardiovasculares Carlos III; Catalan Institute of Nanoscience and Nanotechnology (ICN2); Catalan Institute of Nanoscience and Nanotechnology (ICN2); Institut de Recerca Sant Pau; Institut de Recerca Sant Pau; Hospital de la Santa Creu i Sant Pau; Institut de Recerca Sant Pau; Institut de Recerca Sant Pau; Hospital de la Santa Creu i Sant Pau; Institut de Recerca Sant Pau; Hospital de la Santa Creu i Sant Pau

**Keywords:** Alzheimer's Disease, APOE, Cholesterol, HDL-like lipoprotein

## Abstract

In the central nervous system, apolipoprotein (APO) E-containing high-density lipoprotein (HDL)-like particles mediate the transport of glial-derived cholesterol to neurons, which is essential for neuronal membrane remodeling and maintenance of the myelin sheath. Despite this, the role of HDL-like cholesterol trafficking on Alzheimer’s disease (AD) pathogenesis remains poorly understood. We aimed to examine cholesterol transport via HDL-like particles in cerebrospinal fluid (CSF) of AD patients compared to control individuals. Additionally, we explored the ability of reconstituted HDL containing different APOE isoforms to regulate cholesterol transport.

We evaluated the capacity of CSF HDL-like particles to facilitate radiolabeled unesterified cholesterol efflux from A172 human glioblastoma astrocytes and to deliver cholesterol to SH-SY5Y human neuronal cells. The HDL-like proteome in the AD and control groups was analyzed by liquid chromatography-mass spectrometry (LC-MS/MS). Reconstituted HDL nanoparticles were prepared by combining phospholipids and cholesterol with human APOE3 or APOE4, followed by radiolabeling with unesterified cholesterol.

Our results showed that cholesterol efflux from astrocytes to CSF were similar between AD patients and controls, both under baseline conditions and after activation of ATP-binding cassette transporters A1 and G1. However, CSF HDL-like particle-mediated neuronal cholesterol uptake was significantly reduced in the AD group. LC-MS/MS analysis identified 775 proteins associated with HDL-like particles in both groups, with no major alterations in proteins linked to cholesterol metabolism. However, 27 proteins involved in non-cholesterol-related processes were differentially expressed. Notably, synthetic reconstituted HDL particles containing APOE4 exhibited reduced capacity to deliver cholesterol to neurons compared to those with APOE3.

These findings indicate that CSF HDL-like particles from patients with AD demonstrate impaired cholesterol delivery to neurons. Our study highlights APOE4 as a critical contributor to abnormal neuronal cholesterol uptake in AD pathophysiology.

## Introduction

Alzheimer Disease (AD) is a neurodegenerative disorder that causes difficulty communicating and reasoning, mood changes, and progressive memory loss. Histologically, AD is defined by the pathologic accumulation of extracellular amyloid beta (Aβ) and abnormally hyperphosphorylated intracellular tau filaments in neurons, resulting in amyloid plaques and neurofibrillary tangles respectively, with neuropathological lesions occurring many years before clinical signs [5]. With few effective and approved treatments, AD is a growing public health concern.

The brain is predominantly composed of lipids [3], which are essential for functions such as blood-brain barrier (BBB) regulation, amyloid precursor protein (APP) processing, myelination, membrane remodeling, receptor signaling, oxidation, inflammation, and energy balance [37]. Cholesterol, in particular, is vital for brain health, neuron repair, membrane remodeling, and plasticity [34], and its metabolism has been extensively implicated in the pathogenesis of AD [2, 4, 22].

Since mature lipoproteins containing cholesterol cannot cross the blood brain barrier, cholesterol synthesis in the central nervous system (CNS) primarily relies on astrocytes [26]. Cerebrospinal fluid (CSF), the most nearby biofluid to the brain that can be used to evaluate both normal and aberrant brain physiology, contains unique lipoproteins known as “HDL-like particles” due to their similarity to peripheral high-density lipoproteins (HDL). These particles are present at concentrations 100 times lower than plasma HDL [21], making comprehensive biochemical analysis highly challenging. HDL-like particles are essential for cholesterol transport between glial cells and neurons, with APOE as the primary protein necessary for this process [26]. APOE must be synthesized locally by astrocytes and microglia [26]. Notably, lipidated APOE contributes to the clearance of Aβ peptides, thus potentially impacting their transport in the CNS and contributing to AD pathophysiology [41, 47]. In this context, recent research has confirmed that APOE4 homozygosity is a significant genetic factor contributing to AD [12].

Regarding cholesterol efflux to HDL-like particles in the CNS, ATP binding cassette (ABC) A1 is the main cholesterol efflux transporter in astrocytes, with ABCG1 playing a secondary role [7, 39]. Cholesterol efflux to the CSF is a potential mechanism in AD-related cholesterol metabolism deregulation [27, 48], yet studies using human astrocytes to examine this in AD patients’ CSF are lacking. Concerning cholesterol uptake, HDL-like particles deliver cholesterol to neurons through APOE interaction with specific lipoprotein receptors such as low-density lipoprotein receptor (LDLR), LDLR-related protein (LRP) 1, very-low density lipoprotein receptor, and APOE receptor 2 [6]. In this regard, proprotein convertase subtilisin/kexin type 9 (PCSK9) has been suggested as a potential player, since it degrades some APOE-binding receptors [36].

In this study, we investigated CSF-mediated cholesterol transport between human glioblastoma astrocytes and neurons via HDL-like particles from control individuals and AD patients, examining its association with AD biomarkers and the HDL-like proteome. Additionally, we developed synthetic reconstituted HDL-like nanoparticles containing either APOE3 or APOE4 to assess the impact of the APOE-ε4 isoform on cholesterol transport. Our findings reveal that CSF HDL-like particles from AD patients show reduced efficiency in delivering cholesterol to neurons, with APOE4 emerging as a potential key factor in the disrupted neuronal cholesterol uptake observed in AD pathophysiology.

## Methods

### Human samples

CSF samples were retrospectively selected from the SPIN cohort (Sant Pau Initiative on Neurodegeneration), a multimodal research cohort for biomarker discovery and validation that includes participants with different neurodegenerative dementias, mild cognitive impairment and cognitively normal controls. All participants underwent an extensive neurological and neuropsychological evaluation and had blood extraction and lumbar puncture for CSF AD biomarker analysis as part of their diagnostic work-up [1]. All participants provided written consent to participate in our biomarker program. Further information on the SPIN cohort can be found at https://santpaumemoryunit.com/our-research/spin-cohort.

A total of 20 CSF from control individuals (n = 10) and patients with AD dementia (n = 10) were included. CSF samples were obtained by lumbar puncture under standardized conditions [1].

### CSF AD biomarker analysis

APOE genotype, defined by two single nucleotide polymorphisms, the rs429358 and the rs7412 was performed by Sanger sequencing as previously reported [1]. Brain amyloidosis biomarkers (CSF Aβ_1− 40_, CSF Aβ_1− 42_ and CSF Aβ_42_/Aβ_40_ ratio), tau pathology biomarkers (CSF phosphorylated-Tau181 (p-Tau)) and neurodegeneration biomarkers (CSF total-Tau (t-Tau)) were determined by chemiluminescent immunoassay on a LUMIPULSE G600II automated platform analyzer (Fujirebio®).

### CSF lipid-related parameters measurement

Total CSF cholesterol and free cholesterol were measured by Amplex Red Cholesterol Assay Kit (Thermo Fisher Scientific). APOE, APOJ and APOA1 concentrations were measured by Human ELISA Kits (Thermo Fisher Scientific). PCSK9 concentrations were determined using the Quantikine® ELISA kit (R&D Systems). The kits were designed for plasma samples quantification; therefore, CSF samples dilutions were adapted to the cholesterol, APOs, or PCSK9 concentrations in the CSF.

### Cell culture

Human glioblastoma cells A172 (ATCC® CRL-1620™) were maintained in Dulbecco’s Modified Eagle Medium (DMEM) high glucose with L-glutamine and with sodium pyruvate (Corning) supplemented with 10% fetal bovine serum (FBS) (Pan Biotech) and 100 U/mL penicillin/streptomycin (Dominique Dutscher). Human neuroblastoma cells SH-SY5Y (ATCC® CRL-2266™) were maintained in DMEM high glucose with L-glutamine (Corning) and Ham’s Nutrient Mixture F12 with L-glutamine (Cytiva) 1:1 v/v supplemented with 10% FBS (Pan Biotech) and 100 U/mL penicillin/streptomycin (Dominique Dutscher). Cells were seeded and grown in 75 cm^2^ cell culture flasks and incubated in a humidified incubator (5% CO_2_, 37°C). The medium was renewed every 48 hours and cells were trypsinized once they reached confluence.

Previous to any experiment, human neuroblastoma SH-SY5Y cells were differentiated into functional neurons by replacing maintenance medium with differentiation culture medium for 7 days and refreshment every 72h. Differentiation culture medium consisted of DMEM high glucose with L-glutamine (Corning) and Ham’s Nutrient Mixture F12 with L-glutamine (Cytiva) 1:1 v/v supplemented with 1% FBS, 100 U/mL penicillin/streptomycin and 10 μM retinoic acid (RA), (Sigma-Aldrich/Merck).

### Cholesterol efflux assay

A172 and SH-SY5Y cells were seeded at densities of 50,000 and 100,000 cells per well, respectively, in 24-well plates using their corresponding maintenance culture medium. SH-SY5Y cells were differentiated by incubating them in differentiation culture medium containing RA, as previously described. Twenty-four hours after plating or reaching differentiation, cells were loaded with 0.5 μCi/well of radiolabeled cholesterol ([1α,2α(n)-^3^H] cholesterol, Revvity) added to 5% FBS-supplemented medium. Cells were allowed to capture radiolabeled cholesterol for 48 hours and extensively washed with phosphate-buffered saline (PBS) before performing cholesterol efflux assays. Subsequently, the cells were equilibrated overnight in serum-free medium containing 0.2% free fatty acid bovine serum albumin (Sigma-Aldrich/Merck), either with or without the addition of the ABCA1/G1 activator T0901317 (Cayman Chemicals) at a concentration of 2 μM. Following this, cells were washed and serum-free medium containing either CSF (30% v/v) or reconstituted HDL (5 μg of APOE/mL) was added to the cells. After 4 hours, the medium was collected and 0.1 M NaOH (Sigma-Aldrich/Merck) was added to the cells. The medium fraction was centrifuged for 5 minutes at 250 g to remove floating cells. The cellular fraction was incubated at 4°C with gentle shaking for 48 hours, after which it was collected and sonicated for at least 1 hour. Radiolabeled cholesterol in both medium and cellular fraction was quantified by liquid scintillation counting, and the percentage of cholesterol efflux was calculated by dividing radiolabeled cholesterol in the medium by the sum of radiolabeled cholesterol in the medium and cellular fractions.

### Cholesterol uptake assay

CSF samples or synthetic reconstituted HDL-like nanoparticles were radiolabeled with evaporated [1α,2α(n)-^3^H] cholesterol (0.1 mCi/mL and 0.2mCi/mL respectively) by incubating them overnight at 37°C. The incorporation of radiolabeled cholesterol into HDL-like particles of CSF was verified by isolating HDL-like particles at a density range of 1.063–1.210 g/mL using density gradient ultracentrifugation. A172 and SH-SY5Y cells were seeded at a density of 100,000 cells per well in 24-well plates. Twenty-four hours after plating or reaching differentiation, serum-free medium containing radiolabeled CSF (10% v/v) or reconstituted HDL (5 μg of APOE/mL) was added to the cells. Part of the experiments were conducted in the presence of Human Tau-441/2N4R Protein (ACROBiosystems) or Aβ Protein Fragment _1− 42_ (Sigma-Aldrich/Merck), which were added to the medium at varying concentrations. After 4 hours, the medium was collected and 0.1 M NaOH (Sigma-Aldrich/Merck) was added to the cells. The medium fraction was centrifuged for 5 minutes at 250 g to remove floating cells. The cellular fraction was incubated at 4°C with gentle shaking for 48 hours. Afterwards, it was collected and sonicated for at least 1 hour. Radiolabeled cholesterol in both the medium and cellular fraction was quantified by liquid scintillation counting and the percentage of cholesterol uptake was calculated by dividing radiolabeled cholesterol in the cellular fraction by the sum of radiolabeled cholesterol in the medium and the cellular fraction. Values were normalized to the cellular protein content, determined using the Pierce™ BCA Protein Assay Kit (Thermo Fisher Scientific).

### Quantitative Real Time-PCR

For quantitative Real Time-PCR analyses, A172 and SH-SY5Y cells were seeded at a density of 150,000 cells per well in 12-well plates and cholesterol efflux steps were mimicked. Afterwards, RNA was isolated using the EZ-10 DNAaway RNA Miniprep kit (Bio Basic) and quantified by NanoDrop-2000 spectrophotometry (Thermo Fisher Scientific). cDNA was generated using EasyScript First-Strand cDNA Synthesis SuperMix (Transgen Biotech) and quantitative Real-Time PCR amplification was performed using the GoTaq(R) Probe qPCR Master Mix (Promega). Specific TaqMan probes (Applied Biosystems) were used for *ABCA1* (Hs01059118_m1), *ABCG1* (Hs00245154_m1) and *GAPDH* (Hs02758991_g1), the latter used as internal control gene. Reactions were run on a CFX96TM Real-Time System (Bio-Rad) according to the manufacturer’s instructions. Thermal cycling conditions included 10 minutes at 95°C before the onset of the PCR cycles, which consisted of 40 cycles at 95°C for 15 seconds and at 65°C for 1 minute. The relative mRNA expression levels were calculated using the ΔΔCt method [25].

### Protein staining and Blotting

Plasma HDL was isolated by ultracentrifugation at a density range of 1.063–1.210 g/mL, and the APOA1 concentration was determined using an immunoturbidimetric assay adapted for the COBAS 6000/501c autoanalyzer (Roche Diagnostics). Samples containing plasma HDL or CSF were prepared by adding 40% v/v of 50% m/v sucrose (Sigma-Aldrich/Merck) and 30 μL of each sample was size-separated electrophoretically on 4–15% Mini-PROTEAN® TGX™ Precast Protein Gels (Bio-Rad) at 80 V for 30 minutes, followed by 120 V for 2 hours.

For protein staining, a fixation solution consisting of 40% methanol (Sigma-Aldrich/Merck) and 10% acetic acid (Sigma-Aldrich/Merck) was applied to the gels for 1 hour. Gels were then washed with water and stained overnight with GelCode™ Blue Staining Reagent (Thermo Fisher Scientific). The following day, the gels were washed again with water to remove background staining.

For Blotting, proteins were transferred onto 0.2 μm PVDF membranes (Bio-Rad). The membranes were blocked using EveryBlot Blocking Buffer (Bio-Rad) for 15 minutes and incubated overnight at 4°C with the goat anti-human APOE primary antibody (1:500 dilution, Roche Diagnostics). After incubation, the membranes were washed three times for 10 minutes each with tris-buffered saline containing 0.1% of Tween-20 (TBST) buffer and subsequently incubated with a rabbit anti-Goat IgG HRP-conjugated secondary antibody (ThermoFisher Scientific) for 1 hour. The membranes were washed three times each for 10 minutes with TBST buffer and analyzed using Clarity Western ECL Substrate (Bio-Rad).

Images were captured using a ChemiDoc XRS Gel Documentation System (Bio-Rad) and Image Lab software (version 6.0.1, Bio-Rad).

### Proteomic analysis

CSF samples were size-separated electrophoretically on 4–15% Mini-PROTEAN® TGX™ Precast Protein Gels (Bio-Rad) as described above. After staining, HDL-like particle bands known to contain APOE from Blotting analyses were excised. Each band was placed in an individual tube with acetonitrile (Sigma-Aldrich/Merck) which was removed under vacuum. The bands were stored at −80°C until further analysis.

Bands were rehydrated with 1 mL of bicarbonate 25 mM pH 8.8 and dehydrated with acetonitrile 100%. Protein denaturation was performed incubating the bands with 400 μL of 10 mM DL-Dithiothreitol in 25 mM bicarbonate pH 8.8 for 30 minutes at room temperature (RT) and rotation. For protein alkylation, bands were incubated with 400 μL of 54 mM iodoacetamide in 25 mM bicarbonate pH 8.8 for 45 minutes at RT in rotation and protected from the light. Bands were dehydrated with acetonitrile 100% for 10 minutes and dried in the speed-vacuum. Protein digestion was performed with 1 μg of trypsin per band in 50mM bicarbonate pH 8.8 and acetonitrile 10% buffer overnight at 37°C. Samples were desalted using NestGroup Spin columns following the manufacturer’s instructions, dried in the speed-vacuum and stored at 4°C until liquid chromatography-mass spectrometry (LC-MS/MS) analysis.

LC-MS/MS analysis was conducted using an Evosep One HPLC (Evosep) coupled to an Orbitrap Eclipse Tribrid Mass Spectrometer (Thermo Fisher Scientific) using an Evotip C18 (Evosep) as trapping matrix and an Endurance Evosep (EV1106) column (15 cm x 150 μm ID) coupled to a stainless emitter of 30 μm ID and interfaced with the Mass Spectrometer using Nanospray Flex Ion Source. Column was heated to maintain temperature at 55°C. Peptides were eluted from Evotips and analyzed using Evosep One pre-programmed gradient for 15 samples per day. Samples were analyzed using a data independent acquisition strategy. Full MS resolutions were set to 120,000 at m/z 200 and the full MS AGC target was 300% with a maximum injection time set to Auto. The AGC target value for fragment spectra was set to 1000%. 50 windows of 12 m/z scanning from 400 to 100 m/z were employed with an overlap of 1 Da. MS2 resolution was set to 30,000, IT to 54 ms, and normalized collision energy to 30%. Raw files were analyzed with DIANN (version 1.8.1) using an *in silico* predicted spectral library derived from *Homo sapiens* proteome Uniprot Database (2022 version, containing 20.958 entries). N-terminal excision of methionine and carbamidomethylation of cysteine were set as fixed modifications, while oxidation of methionine was set as a variable modification. The enzyme/cleavage rule was set to Trypsin/P, the digestion type was specific, and a maximum of one missed cleavage per peptide was allowed. Normalization was turned off, and quantification was conducted using Robust LC.

Quantitative information obtained from precursor intensities, as reported by DIANN, was integrated from the precursor level to the peptide level, and then to the protein level, using the WSPP model and Generic Integration Algorithm [14, 32], implemented through the iSanXot program [40, 45]. In this model, quantitative protein values are expressed using the standardized variable Zq (normalized log2-ratios expressed in units of standard deviation based on the estimated variances). For functional analysis, proteins were annotated using DAVID [18].

### Preparation and characterization of synthetic reconstituted APOE3- and APOE4-containing lipoproteins

Synthetic reconstituted HDL particles containing APOE3 or APOE4 (rHDL-APOE3 and rHDL-APOE4) were generated using the cholate-dialysis method [11]. Briefly, the lipid mixture was prepared with 1,2-dimyristoyl-sn-glycero-3-phosphocoline (DMPC, Merck KGaA) and free cholesterol (Merck KGaA) in a chloroform solution at a 9:1 DPMC/free cholesterol molar ratio. Synthetic rHDL-APOE3 and rHDL-APOE4 nanoparticles were fluorescently labeled for specific experiments. To this end, Oregon Green™ 488 1,2-Dihexadecanoyl-sn-Glycero-3-Phosphoethanolamine (Oregon Green™ 488 DHPE, ThermoFisher) were added into the lipid mixture in chloroform solution (0.03 mM). The organic solvent was removed under vacuum and nitrogen to form a dry lipid film, which was then rehydrated with 2 mL PBS containing 60 mg/mL sodium deoxycholate (cholate, Sigma-Aldrich/Merck). This suspension was incubated at 37°C for 30 minutes until a clear solution containing DMPC/free cholesterol/cholate mixed micelles was obtained. For the preparation of synthetic rHDL-APOE3 and rHDL-APOE4 nanoparticles, mixed micelles were incubated with recombinant APOE3 or APOE4 (Thermo Fisher Scientific Inc) at 59:7:1 DMPC/free cholesterol/APOE molar ratio. Next, three incubation cycles were performed at 4°C and 37°C to promote lipid-protein interaction. After incubation, self-assembly of the synthetic rHDL-APOE nanoparticles started with the removal of cholate through extensive dialysis against a 1000-fold excess of PBS at 4°C for 48 hours, using 3,5 kDa Slide-A-Lyzer™ G3 Dialysis Cassettes with two buffer changes. Finally, the dialyzed samples were centrifuged at 16,000 x g for 30 minutes at 4°C to eliminate unbound lipids.

The composition of synthetic rHDL-APOE3 and rHDL-APOE4 nanoparticles, including unesterified cholesterol and APOE, was determined using enzymatic and immunoturbidimetric assays, respectively, with commercial kits adapted for a COBAS 6000 autoanalyzer (Roche Diagnostics and Randox). rHDL-APOE nanoparticles, along with CSF, isolated HDL, and lipid-free APOA1, were size-separated electrophoretically on a 4–15% Mini-PROTEAN® TGX™ Precast Protein Gel (Bio-Rad), followed by protein staining as previously described. Furthermore, particle-size distributions were determined using a dynamic light scattering (DLS) analyser together with non-invasive backscatter technology (Malvern Zetasizer, Malvern Instruments). The morphology of synthetic rHDL-APOE3 and rHDL-APOE4 nanoparticles were also observed by transmission electron microscopy (TEM) with negative staining. An 8 μL aliquot of synthetic rHDL-APOE was added to freshly glow-discharged carbon 300 Mesh copper grids (Ted Pella Inc.) for 1 minute. After blotting excess fluid, samples were stained with 8 μL of 5% uranyl acetate for 1 minute and examined in a JEM 1400 Transmission Electron Microscope (JEOL USA, Peabody).

### Confocal microscopy and flow cytometry

SH-SY5Y neurons were seeded in individual plates of 3.5 cm^2^ (Ibidi®) at a cell density of 50,000 cells per well and differentiated as previously described. Subsequently, 1 mL of serum-free medium with or without synthetic rHDL-APOE3 or rHDL-APOE4 (5 μg/mL), was added to each plate and incubated for 4 hours to facilitate the internalization of synthetic rHDL-APOE nanoparticles. The cells were stained with a solution of 0.5 μL of Hoechst and 1 μL of CellMask™ (Thermo Fisher Scientific) diluted in 1 mL of PBS. Imaging of SH-SY5Y neurons was conducted using a Leica TCS SP5 X Tune confocal microscope (Leica).

The uptake of synthetic rHDL-APOE3 and rHDP-APOE4 nanoparticles labeled with Oregon Green™ 488 DHPE fluorophore by SH-SY5Y cells was analyzed using flow cytometry. SH-SY5Y cells were seeded at a density of 200,000 cells per well in 12-well plates, and the differentiation protocol was performed. Upon reaching differentiation, the cells were incubated with or without synthetic rHDL-APOE3 or rHDL-APOE4 (5 μg/mL) in serum-free medium for 4 hours. After incubation, cells were washed with PBS, trypsinized for 5 minutes at 37°C and resuspended in 100 μL of PBS. Flow cytometry analysis was performed using a MACSQuant Analyzer (Miltenyi Biotec), data were acquired for 10,000 events within the gate representing viable single cells and analyzed using MACSQuant Software.

### Statistical analysis

The number of CSF samples per group was estimated using an α value of 0.05, a power of 80%, and an effect size of 1.28 in the cholesterol efflux and uptake assays. The Shapiro-Wilk normality test was conducted to assess Gaussian distribution. Continuous variables are presented as mean ± SD, while qualitative data are expressed as percentages and analyzed using Fisher’s exact test. The Student’s t-test was used to compare statistical differences between AD and control groups. One-way ANOVA, followed by a post-test for linear trend, was used to evaluate relative cholesterol uptake in human neuroblastoma cells, mediated by control CSF HDL-like particles, in the absence or presence of t-Tau or Aβ_1−42_. Associations between variables were assessed using Pearson’s correlation coefficient. The statistical analyses were conducted using GraphPad Prism 8 (GraphPad, San Diego, CA, USA). Differential expression analysis for proteomics data was performed using limma moderated t-statistics. A p-value of less than 0.05 was considered statistically significant.

## Results

### Cerebrospinal Fluid Cholesterol and Apolipoprotein Levels Are Similar in Patients with AD and Control Individuals

CSF AD biomarkers and CSF cholesterol and APO levels are shown in Table 1. As expected, AD patients had significantly higher CSF concentrations of t-Tau and p-Tau, as well as decreased levels of Aβ_1−42_ and a reduced Aβ_1−42/Aβ1−40_ ratio compared to control individuals. Regarding CSF total and free cholesterol, APO (APOE, APOJ and APOA1), and PCSK9 concentrations, no significant differences were observed between the groups. The frequency of APOE4 carriers was significantly higher among AD patients (7 APOE-ε4/ε3, 1 APOE-ε4/ε4, and 3 APOE-ε3/ε3), whereas all control individuals were APOE-ε3/ε3.

### Astrocyte Cholesterol Efflux to Cerebrospinal Fluid is Similar in Patients with AD and Control Individuals

**Figure 1:**
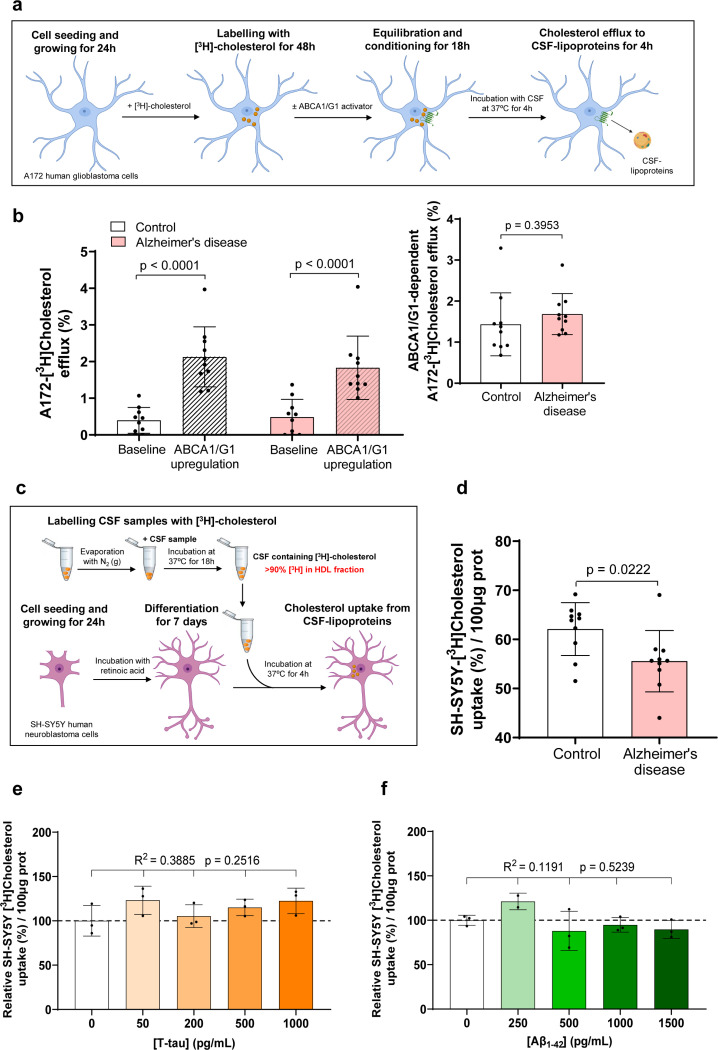
Astrocyte Cholesterol Efflux to CSF Remains Similar in AD and Control Groups, whereas CSF HDL-like-mediated Cholesterol Delivery to Neurons is Impaired in AD. (a) Astrocyte Cholesterol Efflux Assay. Human glioblastoma astrocytes were cultured for 24 hours, followed by a 48-hour incubation with radiolabeled cholesterol. Cells were then treated for 18 hours with or without T0901317 to activate ABCA1/G1 pathways. Serum-free medium containing CSF was added for 4 hours. Both the medium and cell fractions were processed to quantify radiolabeled cholesterol. (b) Astrocyte Cholesterol Efflux Results: Left panel - Cholesterol efflux from human glioblastoma astrocytes to CSF (30% v/v) is shown for both control and AD samples, under baseline conditions and following T0901317 pre-treatment. Right panel - Specific ABCA1/G1-dependent cholesterol efflux was calculated subtracting baseline levels from those observed in ABCA1/G1-expressing cells. (c) CSF HDL-like-Mediated Cholesterol Uptake Assay: Human neuroblastoma cells were seeded and differentiated into neurons in a low-serum medium containing retinoic acid. After 24 hours, radiolabeled CSF HDL-like particles containing unesterified cholesterol were added (10% v/v). The cells were incubated with serum-free medium containing CSF for 4 hours, and both the medium and cell lysates were processed for radiolabeled cholesterol quantification. (d) Cholesterol Uptake results: CSF HDL-like-mediated cholesterol uptake was measured in human neurons exposed to CSF from both control individuals and patients with AD. (e and f) Influence of Tau and Aβ_1–42_ on Neuronal Cholesterol Uptake: Cholesterol uptake in SH-SY5Y neurons mediated by control CSF HDL-like particles was assessed in the presence of Tau or Aβ_1–42_ added to the culture media at concentrations up to 1,000 and 1,500 pg/mL, respectively. Values are shown as the mean ± SD for 10 subjects per group in panels (b) and (d). The Student’s t-test was used to compare the CSF HDL-like-mediated cholesterol uptake by neurons, as well as the astrocyte cholesterol efflux under various conditions between the control and AD groups. One-way ANOVA with a post-test for linear trend was used in panels (e) and (f). Three separate experiments were carried out for each condition.

We adapted a cholesterol efflux assay by loading both A172 human glioblastoma astrocytes and differentiated SH-SY5Y human neurons with radiolabeled unesterified cholesterol and evaluated the release rate of radiolabeled cholesterol to CSF (see Fig. 1a for a schematic diagram of the method involving astrocytes). Cells were also treated with an LXR agonist to compare their responses to ABCA1/G1-mediated cholesterol efflux induction. Under baseline conditions, both cell lines exhibited low cholesterol efflux percentages that were similar to control CSF. However, LXR agonist treatment significantly increased cholesterol efflux in astrocytes, while it remained unchanged in neurons (Supplementary Fig. S1a). Consistent with the cholesterol efflux assay results, both ABCA1 and ABCG1 gene expression were highly upregulated upon LXR agonist exposure in astrocytes. In contrast, these changes were less pronounced in neurons (Supplementary Fig. S1b and S1c).

Next, we evaluated cholesterol efflux from astrocytes to CSF samples obtained from AD patients and compared the percentage of efflux with that of control individuals under baseline conditions and after pre-treating the cells with an LXR agonist. The LXR agonist induced an increase in CSF-mediated cholesterol efflux compared to baseline conditions (Fig. 1b **left panel**). However, CSF-mediated cholesterol efflux was similar when induced by CSF samples from both patients with AD and control individuals, under baseline conditions and after ABCA1/G1 activation. Furthermore, when comparing ABCA1/G1-dependent cholesterol efflux between groups (calculated as the increase in efflux after subtracting baseline levels), CSF from AD patients and control individuals induced cholesterol efflux at similar levels (Fig. 1b **right panel**).

### Cerebrospinal Fluid Lipoprotein-Mediated Cholesterol Delivery to Neurons Is Impaired in Patients with AD

We developed a cholesterol uptake assay by incorporating radiolabeled unesterified cholesterol into the HDL-like particles of CSF and evaluated the rate of radiolabeled cholesterol uptake in both A172 human glioblastoma astrocytes and differentiated SH-SY5Y human neurons (see Fig. 1c for a schematic diagram of the method involving neurons). In order to set up the best uptake condition, neurons were incubated with radiolabeled control CSF during 2, 4 and 8 hours. The percentage of CSF-mediated cholesterol uptake showed a linear time-dependent increase (Supplementary Fig. S2a). Next, the uptake cholesterol assay was performed simultaneously in astrocytes and differentiated neurons, resulting in an increased percentage of radiolabeled cholesterol uptake within the 4-hour period in neurons when compared to astrocytes (Supplementary Fig. S2b), indicating that neurons may rely more heavily on external cholesterol uptake as a resource.

To evaluate cholesterol delivery to neurons, we then compared CSF HDL-like-mediated cholesterol uptake in differentiated SH-SY5Y human neurons from CSF samples acquired from control individuals and patients with AD. The percentage of radiolabeled cholesterol uptake within a 4-hour period was significantly reduced in neurons incubated with CSF from AD patients (Fig. 1d). Given that Aβ and Tau may potentially alter the lipid-binding function of HDL-like particles [9, 17, 43], we examined the association between CSF-mediated cholesterol uptake and other CSF parameters (Table 2). We observed a positive trend between CSF-mediated cholesterol uptake and CSF Aβ_1−42_ levels; however, this correlation did not reach statistical significance (Table 2). To further investigate whether Aβ_1−42_ or even Tau could directly alter CSF-mediated cholesterol uptake in neurons, we added isolated Aβ_1−42_ or Tau to CSF in the presence of differentiated neurons at increasing concentrations. Neither Tau nor Aβ_1−42_ affected the percentage of radiolabeled cholesterol uptake in neurons within the physiological range (Fig. 1e and 1f).

### The Cerebrospinal Fluid HDL-like Proteome Is Modified in Patients with AD

**Figure 2:**
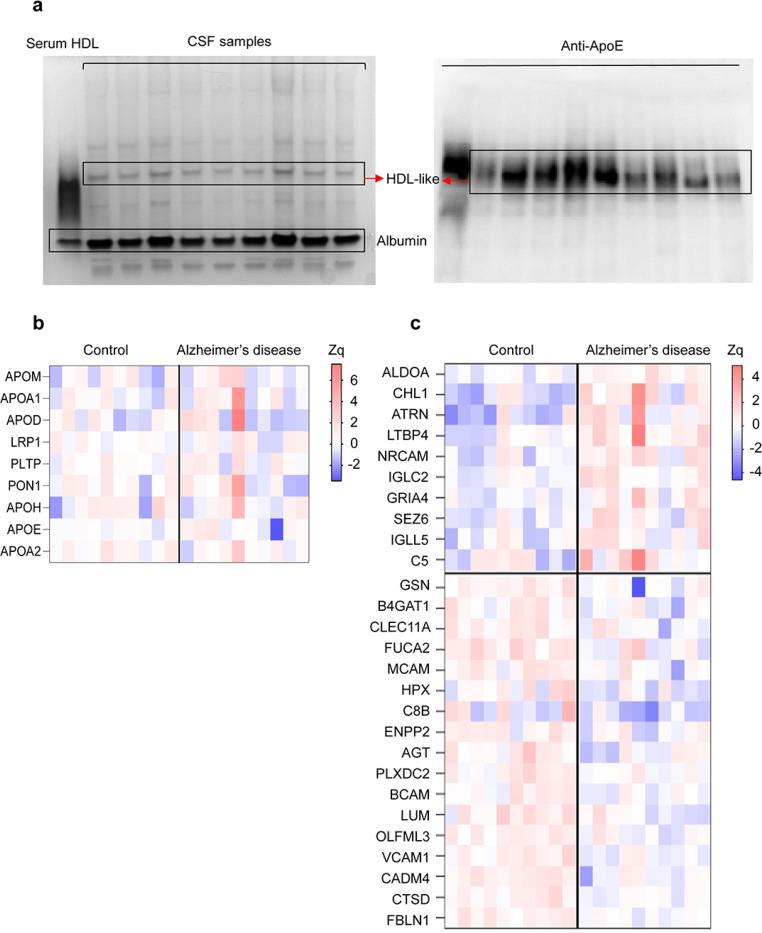
Altered Proteome of CSF HDL-like Particles in AD. (a) Native Gel Electrophoresis of CSF Samples. Representative native polyacrylamide gel electrophoresis of CSF from patients with AD and control individuals. Left panel - HDL-like particles and albumin bands following Coomassie blue staining. Right panel - Corresponding nitrocellulose blot probed with a polyclonal anti-APOE antibody, identifying the HDL-like band. (b) Proteins from Cholesterol Metabolism Quantified in HDL-like Particles from CSF. Increased (red) or decreased (blue) abundances are shown according to the indicated Zq scale. APOM: Apolipoprotein M, APOA1: Apolipoprotein A-I, APOD: Apolipoprotein D, LRP1: Low-density lipoprotein receptor-related protein 1, PLTP: Phospholipid transfer protein, PON1: Serum paraoxonase/arylesterase 1, APOH: Beta-2-glycoprotein 1, APOE: Apolipoprotein E, APOA2: Apolipoprotein A-II. (c) Differentially Regulated Proteins in HDL-like Particles from the CSF of Patients with AD. Heat map depicting significant protein abundance changes (p < 0.05) in AD and control groups. Increased (red) or decreased (blue) abundances are shown according to the indicated Zq scale. Differential protein expression analysis was performed using moderated t-statistics (limma test). ALDOA: Fructose-bisphosphate aldolase A, ACHL1: Neural cell adhesion molecule L1-like protein, ATRN: Attractin, LTBP4: Latent-transforming growth factor beta-binding protein 4, NRCAM: Neuronal cell adhesion molecule, IGLC2: Immunoglobulin lambda constant 2, GRIA4: Glutamate receptor 4, SEZ6: Seizure protein 6 homolog, IGLL5: Immunoglobulin lambda-like polypeptide 5, C5: Complement C5, GELS: Gelsolin, B4GAT1: Beta-1,4-glucoronyltransferase 1, CLEC11A: C-type lectin domain family 11 member A, FUCA2: Plasma alpha-L-fucosidase, MCAM: Cell surface glycoprotein MUC18, HPX: Hemopexin, C8B: Complement component C8 beta chain, ENPP2: Ectonucleotide pyrophosphatase/phosphodiesterase family member 2, AGT: Angiotensinogen, PLXDC2: Plexin domain-containing protein 2, BCAM: Basal cell adhesion molecule, LUM: Lumican, OLFML3: Isoform 2 of Olfactomedin-like protein 3, VCAM1: Vascular cell adhesion protein 1, CADM4: Cell adhesion molecule 4, CTSD: Cathepsin D, FBLN1: Fibulin-1.

We separated CSF lipoproteins according to size using nondenaturing polyacrylamide gradient gel electrophoresis. Native polyacrylamide gradient gel electrophoresis illustrated a homogeneous band for large HDL-like size (Fig. 2a **left panel**). Western blot analyses of the gel revealed that APOE was located at the same height as the HDL-like particles band (Fig. 2a **right panel**). Next, we separated the CSF lipoproteins from the CSF of patients with AD and control individuals with the polyacrylamide gradient gel electrophoresis, and performed a comparative MS profiling of proteins differentially expressed in the HDL-like particles band. A total of 775 proteins were quantified, from which 239 were detected with two or more peptides and were consistently present across all CSF samples (Supplementary Table S1). Functional analysis revealed that the majority of these proteins were mainly involved in blood coagulation, carbohydrate and cholesterol metabolism, cell adhesion and migration, complement activation, oxidative stress response, developmental processes, and proteolysis (Supplementary Fig. S3). A total of 24 proteins were related to cholesterol metabolism, but only 9 were detected in all CSF samples. APOE was the most abundant protein in HDL-like particles. However, none of the cholesterol-related proteins showed significant differences between the control and AD groups (Fig. 2b). In contrast, the relative abundance of 27 non-cholesterol-related proteins changed significantly in patients with AD compared to controls, with 9 up-regulated and 18 down-regulated (Fig. 2c). These proteins were involved in several biological processes, including cell adhesion, complement activation, oxidative stress, and proteolysis. Interestingly, some of the up- and down-regulated proteins, such as ALDOA, NRCAM, FDLN1 and CTSD, have been described to be related to AD pathogenesis.

### APOE4-Containing HDL Exhibits Impaired Cholesterol Delivery to Neurons

**Figure 3:**
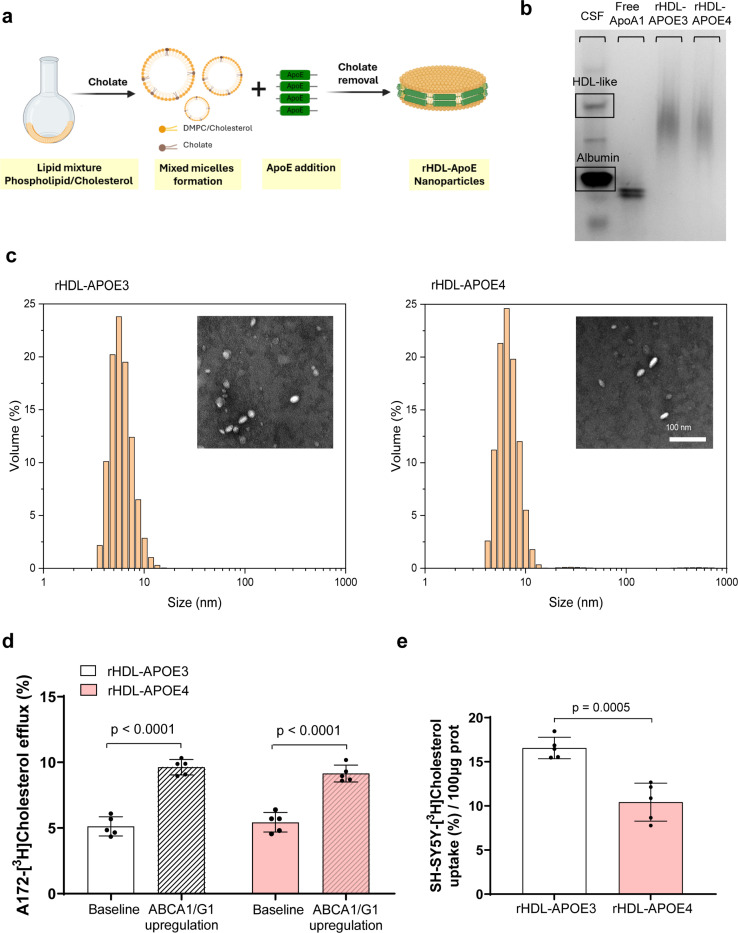
Synthetic Reconstituted HDL Nanoparticles Containing Apolipoprotein E4 Exhibit Impaired CholesterolDelivery to Neurons. (a) Schematic Representation of the Synthesis of Reconstituted (r)HDL-APOE Nanoparticles: Recombinant APOE3 or APOE4 were combined with DMPC and cholesterol in a molar ratio of 59:7:1. The mixture underwent three cycles of vortexing and temperature modulation, alternating between 37°C and 4°C, to optimize APOE-lipid interactions. (b) Native Gel Electrophoresis of Synthetic rHDL-APOE3 and HDL-APOE4 Nanoparticles: Representative native polyacrylamide gel electrophoresis image of synthetic rHDL-APOE3 and rHDL-APOE4 nanoparticles, visualized with Coomassie Blue staining. (c) Characterization of Synthetic rHDL-APOE3 and HDL-APOE4 Nanoparticles: The particle size distributions of purified synthetic rHDL-APOE3 and rHDL-APOE4 nanoparticles were analyzed using dynamic light scattering. Representative images from TEM are also shown as insets. (d) Astrocyte Cholesterol Efflux to Synthetic rHDL-APOE3 and rHDL-APOE4: Cholesterol efflux from human glioblastoma astrocytes to synthetic rHDL-APOE3 and rHDL-APOE4 (5 μg/mL) was measured under baseline conditions and after T0901317 pre-treatment, as described in Fig. 1A. (e) Neuronal Cholesterol Uptake Mediated by Synthetic rHDL-APOE3 and rHDL-APOE4: synthetic rHDL-APOE3 and rHDL-APOE4 (5 μg/mL) were loaded with radiolabeled unesterified cholesterol, and their capacity to facilitate cholesterol uptake in human neuroblastoma cells was assessed as described in Fig. 1c. Mean ± SD is used to express values. Student t-tests were used to compare HDL-mediated neuronal cholesterol delivery between synthetic rHDL-APOE3 and rHDL-APOE4, as well as astrocyte cholesterol efflux under various conditions. Five separate experiments were conducted to evaluate each condition.

Given the higher prevalence of APOE4 carriers among patients with AD, we prepared both synthetic rHDL-APOE3 and HDL-APOE4 nanoparticles by assembling phospholipids and cholesterol with recombinant human APOE3 or APOE4 (see Fig. 3a for a schematic diagram of their synthesis). Both synthetic rHDL-APOE3 and rHDL-APOE4 nanoparticles showed a similar HDL migration pattern (Fig. 3b) and size (Fig. 3c), as well as a similar cholesterol/APOE ratio (0.278 for synthetic rHDL-APOE3 and 0.287 for rHDL-APOE4).

We then mimicked cholesterol efflux from astrocytes by replacing the AD and control CSFs with synthetic rHDL-APOE3 and rHDL-APOE4 nanoparticles, following the method depicted in Fig. 1a. When comparing cholesterol efflux between the two rHDL nanoparticles, both synthetic rHDL-APOE3 and rHDL-APOE4 induced cholesterol efflux from astrocytes at similar levels. (Fig. 3d). We also compared the ability of both synthetic rHDLs, which were radiolabeled as in the graphic depicted in Fig. 1c, to mediate cholesterol uptake in neurons. The percentage of radiolabeled cholesterol uptake was significantly reduced in neurons incubated with synthetic rHDL-APOE4 compared to rHDL-APOE3 (Fig. 3e). We further evaluated the capacity of neurons to internalize an Oregon Green–labeled phospholipid incorporated into synthetic rHDL-APOE3 and rHDL-APOE4 nanoparticles using confocal microscopy and a flow cytometry assay. After a 4-hour incubation with the fluorescently labeled nanoparticles, SH-SY5Y neurons exhibited a trend toward decreased internalization of rHDL-APOE4-associated phospholipids compared with rHDL-APOE3 (Supplementary Fig. S4).

## Discussion

In this study, we aimed to assess the ability of HDL-like particles from AD patients’ CSF to promote cholesterol efflux from human glioblastoma astrocytes and evaluate their capacity to deliver cholesterol to human neurons. Previous studies have reported mixed results regarding APOE levels in AD [10, 13, 16, 19, 27, 29, 42, 48]. In our study, CSF APOE levels were similar between the AD and control groups and were unaffected by sex distribution. Additionally, the levels of CSF APOA1 and cholesterol were similar between patients with AD and control subjects. Thus, our results indicate that the presence of the APOE-ε4 allele in patients with AD does not influence the overall levels of the main CSF HDL-like lipid and protein components. Astrocyte cholesterol efflux to CSF HDL-like lipoproteins containing APOE is a complex process involving both passive diffusion—driven by concentration gradient—and active energy-dependent pathways mediated by ABCA1 and ABCG1 transporters [7, 8]. We evaluated the ability of human A172 glioblastoma astrocytes and SH-SY5Y neurons to mediate cholesterol efflux to CSF under basal conditions and following stimulation of the ABCA1- and ABCG1-dependent pathways. Consistent with previous findings [8], human A172 glioblastoma astrocytes showed a higher capacity for inducing ABCA1/G1-mediated cholesterol efflux to CSF compared to neurons. Notably, astrocyte cholesterol efflux rates to CSF were similar between AD and control groups, both under baseline conditions and after activating of the ABCA1/G1-dependent pathway. This outcome aligns with the findings of an earlier report [10], which observed comparable cholesterol efflux to CSF from rat astrocytes incubated under baseline conditions in both AD and control samples. Conversely, another study found reduced cholesterol efflux from human glioblastoma U373-MG astrocytoma cells to AD CSF compared to control CSF after ABCA1/G1-dependent pathway stimulation [46]. The higher CSF APOE levels in AD patients in this latter study might explain the reduced capacity of AD CSF to promote astrocyte cholesterol efflux, similar to the effect observed with macrophages [35]. It should be noted that human astrocyte models with the APOE4 genotype have demonstrated reduced cholesterol efflux to plasma HDL, primarily due to APOE4’s negative impact on ABCA1 expression at the cell membrane [39, 44]. Indeed, Human induced pluripotent stem cell-derived astrocytes secrete abundant APOE lipoprotein particles, with APOE4 particles transporting fewer lipids compared to APOE3 particles [49]. In our study, however, we used human glioblastoma astrocytes with identical genetic material to specifically assess potential differences in the ability of CSF HDL-like to promote cellular cholesterol efflux, thereby minimizing any confounding effects from cellular APOE variations.

To study cholesterol delivery, we developed an isotopic cholesterol uptake assay by incorporating radiolabeled unesterified cholesterol into the HDL-like particles of CSF to evaluate the ability of human A172 glioblastoma astrocytes and SH-SY5Y neurons to take up cholesterol from CSF. The high cholesterol uptake rates observed in human SH-SY5Y neurons confirmed their suitability as a model for cholesterol uptake assays. Our results also revealed that, compared to controls, cholesterol uptake in human neurons was significantly reduced when mediated by CSF from patients with AD, indicating a diminished capacity of neurons to acquire cholesterol from HDL-like particles in AD CSF. Although CSF levels of PCSK9—the primary protein responsible for degrading APOE-binding receptors—have been reported as elevated in patients with AD in one study [50], we observed no differences in CSF PCSK9 concentrations between AD and control groups. Thus, the role of PCSK9 in AD remains unclear and warrants further investigation.

Although several studies have investigated the protein composition of CSF, only one recent study has specifically analyzed the lipoprotein proteome in CSF from control individuals, utilizing fluorescent high-resolution size-exclusion chromatography fractionation [30]—though this required a substantial CSF volume—. In our study, we characterized the proteome of HDL-like particles in CSF samples from both patients with AD and control individuals by analyzing a homogeneous HDL-like particles size band isolated using native polyacrylamide gradient gel electrophoresis. We identified a total of 775 proteins associated with HDL-like particles across both groups. Regarding cholesterol metabolism-related proteins, nine were consistently present in all CSF samples, with no significant quantitative differences between AD and control groups. The lack of differences in CSF HDL-like-associated APOE levels aligns with our CSF biochemical analyses and those of Martinez-Morillo *et al*. [28], who also observed no difference in CSF APOE levels between patients with AD and non-AD patients when measured by mass spectrometry. We also identified 27 non-cholesterol metabolism-related proteins that were expressed differently in AD and control groups. Many of these proteins were linked to cell adhesion, complement activation, oxidative stress, and proteolysis. Among the proteins upregulated in the HDL-like particles from patients with AD, aldolase A (ALDOA) and neuronal cell adhesion molecule (NrCAM) are particularly noteworthy. ALDOA, a glycolytic enzyme, is elevated in the CSF of patients with AD and has been linked to impaired glucose metabolism in the AD brain [15]. NrCAM, a synaptic cell adhesion molecule, is crucial for synaptic plasticity and has been implicated in AD pathology via its interactions with Aβ and its impact on synapse function and integrity [23]. Among the downregulated proteins, fibulin-1 (FBLN1) and cathepsin D (CTSD) are of particular interest. FBLN1 binds to the N-terminal domain of APP, modulating its neurotrophic activity [33], while CTSD, a lysosomal protease, plays a role in the autophagy-lysosomal Aβ clearance pathway [20]. It remains unclear whether the association of these proteins with HDL-like particles in CSF affects their functions in neuroinflammation, complement activation, neurotrophic activity, and amyloid-beta toxicity, warranting further investigation into their potential roles.

Since our biochemical and proteomic analyses revealed no differences in CSF HDL-like proteins involved in cholesterol transport, we investigated whether the observed alterations in cholesterol transport could arise from functional differences among the APOE isoforms. Previous studies have reported conflicting findings on the lipidation of APOE isoforms in astrocytes. One study observed similar ABCA1-dependent cholesterol and phosphatidylcholine efflux from astrocytes to recombinant lipid-free APOE regardless of the APOE isoform [24], while another study found that recombinant lipid-free APOE3 facilitated greater cholesterol efflux compared to APOE4 [31]. These latter findings align with the lower ABCA1 expression and reduced cholesterol efflux observed in rodent primary astrocytes incubated with recombinant APOE4 [39]. In our study, we directly evaluated the ability of synthetic reconstituted HDL-like nanoparticles containing either recombinant APOE3 or APOE4 isoforms to promote both cholesterol efflux from human A172 glioblastoma astrocytes and cholesterol uptake by SH-SY5Y neurons. Our results showed no significant differences in cholesterol efflux between synthetic rHDL-APOE3 and HDL-APOE4. However, in the cholesterol uptake assay, SH-SY5Y neurons exhibited a significantly reduced capacity to internalize radiolabeled cholesterol when mediated by synthetic rHDL-APOE4 compared to rHDL-APOE3. This suggests that the APOE4 isoform may have a lower affinity or weaker interaction with neuronal APOE-binding receptors, impairing neuronal cholesterol delivery. Supporting these findings, a previous study using hippocampal rat neurons also demonstrated that cholesterol uptake is dependent on the APOE isoform, with reduced radiolabeled cholesterol delivery to rat neurons when bound to lipidated APOE4 isoform [38].

As a limitation of this study, we must consider that the cholesterol export activity of glioblastoma astrocytes may be exceptionally high and significantly different from that of astrocytes in situ in the adult or aged brain. While the statistical power calculations indicated that a sample size of ten per group would provide sufficient sensitivity to detect changes, the sample size limited our ability to compare the APOE-ε4 versus APOE-ε3 genotypes among the AD samples. Additionally, the control and AD groups were not perfectly matched for age and sex, with fewer females in the control group. Nevertheless, variations in age and sex distribution did not appear to influence CSF HDL-like-mediated cholesterol delivery to neurons.

In conclusion, our findings indicate that cholesterol transport from CSF HDL-like particles into neurons is impaired in AD patients. Biochemical and proteomic analysis of CSF HDL-like particles showed no significant differences in cholesterol metabolism-related proteins between patients with AD and controls, and neither Tau nor Aβ was found to interfere with this process. Notably, neurons show impaired uptake of APOE4 associated rHDL-like particles compared to APOE3. Future prospective studies are needed to establish whether impaired neuronal cholesterol delivery directly contributes to AD progression.

## Supplementary Material

Supplement 1

## Figures and Tables

**Table 1:** Population Summary and CSF Parameters from Control Individuals (n = 10) and Patients with AD (n = 10).

	Control (n = 10)	AD (n = 10)	p-value
Biological sex (M/F)	8/2	4/6	-
Age (years)	68.6 ± 3.1	72.3 ± 2.7	0.0108
APOE-ε4 carriers	0/10	7/10	0.0031
CSF parameters			
Aβ_1−42_ (pg/mL)	1198.0 ± 330.2	479.1 ± 117.1	< 0.0001
Aβ_1−40_ (pg/mL)	11762.4 ± 2730.6	11041.3 ± 2615.8	0.5540
Aβ_1−42_/Aβ_1−40_ ratio	0.10 ± 0.01	0.04 ± 0.01	< 0.0001
T-tau (pg/mL)	258.5 ± 52.3	763.4 ± 429.82	0.0017
P-tau (pg/mL)	36.3 ± 6.7	121.15 ± 62.8	0.0005
Total cholesterol (μg/mL)	3.23 ± 0.71	3.21 ± 1.27	0.7936
Free cholesterol (μg/mL)	1.63 ± 0.35	1.67 ± 0.83	0.9116
APOA-I (μg/mL)	0.60 ± 1.01	0.34 ± 0.52	0.4763
APOE (μg/mL)	17.3 ± 9.2	16.5 ± 7.2	0.8305
APOJ (μg/mL)	10.1 ± 2.2	10.7 ± 4.0	0.6662
PCSK9 (ng/mL)	3.23 ± 1.08	3.18 ± 1.56	0.9423

Values are n or mean ± SD. The Shapiro-Wilk normality test was conducted to assess Gaussian distribution. Unpaired t-tests were performed for all parameters. Qualitative data are expressed as percentages and analyzed using Fisher’s exact test.

**Table 2:** Association of Neuronal CSF-mediated Cholesterol Uptake with CSF Biochemical Parameters

	Pearson r	p-value
Aβ_1−42_ (pg/mL)	0.4081	0.0741
Aβ_1−40_ (pg/mL)	0.2206	0.3499
Aβ_1−42/Aβ1−40_ ratio	0.4359	0.0547
T-tau (pg/mL)	− 0.0145	0.9516
P-tau (pg/mL)	− 0.0960	0.6871
Total cholesterol (μg/mL)	− 0.1300	0.5847
Free cholesterol (μg/mL)	0.2029	0.3908
APOA-I (μg/mL)	0.0601	0.8014
APOE (μg/mL)	− 0.1484	0.5322
APOJ (μg/mL)	0.1086	0.6485
PCSK9 (ng/mL)	− 0.1105	0.6429

Associations between variables were assessed using Pearson’s correlation coefficient.

## Data Availability

The data, analytical methods, and study materials will be available to other researchers for purposes of reproducing the results or replicating the procedure upon reasonable request. Source data are provided with this paper. The mass spectrometry proteomics data have been deposited to the ProteomeXchange Consortium via the PRIDE partner repository with the dataset identifier PXD057614.
