# Supplementary material for: Impaired Cerebrospinal Fluid Lipoprotein-Mediated Cholesterol Delivery to Neurons in Alzheimer’s Disease
Source: Res Sq. 2024 Dec 23:rs.3.rs-5682870. Preprint. [Version 1] doi: 10.21203/rs.3.rs-5682870/v1 (PMC11703344; doi:10.21203/rs.3.rs-5682870/v1)
Supplement: Supplement 1 [file NIHPPRS5682870V1-supplement-1.pdf]

Supplementary Files

This is a list of supplementary files associated with this preprint. Click to download.

- SupplementaryMaterial12192024.pdf
